# Distance doesn't matter: migration strategy in a seabird has no effect on survival or reproduction

**DOI:** 10.1098/rspb.2022.2408

**Published:** 2023-04-26

**Authors:** Rosemarie Kentie, J. Morgan Brown, Kees C. J. Camphuysen, Judy Shamoun-Baranes

**Affiliations:** ^1^ Institute for Biodiversity and Ecosystem Dynamics, University of Amsterdam, Amsterdam 1012 WX, The Netherlands; ^2^ Department of Coastal Systems, NIOZ Royal Netherlands Institute for Sea Research, Den Burg, Texel 1797 SZ, The Netherlands

**Keywords:** migration, carry-over effects, pre-breeding period, reproduction, egg volumes, seasonal survival

## Abstract

Migrating animals show remarkable diversity in migration strategies, even between individuals from the same population. Migrating longer distances is usually expected to be costlier in terms of time, energy expenditure and risks with potential repercussions for subsequent stages within the annual cycle. Such costs are expected to be balanced by increased survival, for example due to higher quality wintering areas or lower energy expenditure at lower latitudes. We compared reproductive parameters and apparent survival of lesser black-backed gulls (*Larus fuscus*) breeding in The Netherlands, whose winter range extends from the UK to West Africa, resulting in one-way migration distances that differ by more than 4500 km. Individuals migrating furthest arrived later in the colony than shorter distance migrants, but still laid in synchrony with the colony and consequently had a shorter pre-laying period. This shorter pre-laying period affected neither egg volumes nor hatching success. We found no relationship between migration distance and apparent survival probability, corresponding with previous research showing that annual energy expenditure and distance travelled throughout the year is similar across migration strategies. Combined, our results indicate an equal fitness payoff across migration strategies, suggesting there is no strong selective pressure acting on migration strategy within this population.

## 1. Introduction

Seasonal migration is a life-history strategy that enables animals to exploit peaks in resource abundance in seasonal environments for breeding while avoiding deteriorating environmental conditions during other parts of the year [1,2]. Migration has evolved in a wide range of animal taxa [3], but is epitomized by avian migrants whose capacity for flight allows them to efficiently travel thousands of kilometres [4,5]. There is remarkable variation in migration patterns among species, but also among individuals from the same population. In extreme cases, wintering regions can span entire flyways [6–9], leading to inter-individual variation in both distance travelled during migration, as well as conditions experienced on wintering areas, creating different ‘migration strategies'.

The migratory periods are generally assumed to be costly, in terms of time, energy and mortality risk [2,10,11], and these costs are thought to increase with migration distance. Costs may be experienced directly during migration (i.e. mortality [12–14]), or be carried over to non-migratory periods (i.e. later arrival dates, lower reproductive success [15,16]). Longer-distance migrants are often associated with later arrival dates [17–19], because of the time it takes to travel or to refuel along the way [20]. Arriving late could be a disadvantage, as earlier-arriving individuals have priority access to the highest-quality territories and mates [21–24]. Furthermore, assuming that individuals need time to refill their energy levels and settle before breeding, earlier-arriving individuals are able to start breeding earlier, often resulting in higher reproductive success [25–28].

Costs of migration may be compensated for by better environmental conditions in non-breeding areas at lower latitudes. For example, maintenance metabolism is expected to be lower due to milder weather conditions, and availability and reliability of resources is expected to be higher compared to resources closer to the breeding ground [6,10,29–31]. Thus, more distant wintering areas may provide overall improved survival probability counterbalancing potentially reduced reproductive performance, which could explain why different migration strategies have emerged [1,2,29].

Lesser black-backed gulls (*Larus fuscus)* that breed in northwest Europe migrate to diverse winter areas between the UK and West Africa—a 4500 km one-way difference [9,19]. Our studies have shown that individuals are consistent in their migration strategy [19], and migration distance is not associated with sex nor size of the individual [9]. Longer-distance migrants return later to the breeding colony compared to shorter distance migrants [9,19]. Individuals wintering furthest south have a period of concentrated energy expenditure during spring migration, which could pose a mortality risk or influence their body condition upon arrival [32]. Yet, on an annual basis, energy expenditure is similar among migration strategies [32].

In this study, we used a combination of colour-ring resightings and GPS tracking data to investigate whether migration strategy relates to the time between arrival and laying (the pre-laying period), reproduction and survival of lesser black-backed gulls breeding in The Netherlands. We hypothesized that a minimum pre-laying period is required prior to laying, and thus late-arriving longer-distance migrants start breeding after the laying peak. Laying late has repercussions on breeding success [33]; therefore, we expect that longer-distance migrants have lower hatching success. Alternatively, a shorter pre-laying period may result in smaller egg sizes, a larger egg size variation or lower hatching success, because longer-distance migrants had less time to regain body condition and may have territories or partners of lower quality. Following this, if fitness costs are to be balanced across migration strategies, we anticipate annual survival to increase with migration distance (e.g. if more distant wintering areas offer more beneficial environmental conditions [1,2,29]). Alternatively, concentrated peaks in energy expenditure during spring migration may present an increased risk to survival that increases with migration distance, suggesting unequal fitness among strategies.

## 2. Methods

### (a) Marking individuals

Gulls were individually marked during their breeding season in two mixed herring gull (*L. argentatus*) and lesser black-backed gull colonies: between 2006 and 2020 in a coastal dune area on the Wadden Sea island Texel (‘Texel’, 53°01'N, 04°43'E), and between 2008 and 2020 on an artificial island in the mouth of the IJmuiden harbour (‘IJmuiden’, 52°28'N, 04°34'E). Adults were captured during the incubation phase using walk-in traps (May–June), and nearly fledged chicks were hand caught after the chick rearing phase (June–July). Gulls were marked with a numbered steel ring and a green colour ring with an engraved unique four-letter combination. Sex was assessed for adults using head and bill measurements [33]. Targeted resighting effort was carried out during the breeding season in both colonies by ourselves and a team of dedicated volunteers, while winter resightings were largely reported by citizen scientists.

Between 2008 and 2020, a subset of adult birds (*n* = 139) were additionally fitted with solar-powered GPS trackers (12.5–18 g UvA Bird Tracking System [34]) using a backpack harness [35]. GPS trackers were below 3% of body weight, see Camphuysen *et al*. [36] for more details on capture and tagging methods. Outside the breeding season, GPS locations were generally taken every 20 min and stored data were downloaded remotely once birds returned to the colony. The first date a bird returned within 3 km of the colony in spring was determined as its arrival date. GPS-tagged birds were included in reproductive analyses, including data from the year they were tagged, as previous research has shown that GPS tagging does not influence breeding success [37]. Earlier work also did not find an effect of GPS tracking on return rates [35], so tagged birds were included in survival analyses. However, since this previous work did not include winter location in the estimate of return rates, we also report results of survival models excluding tagged birds in electronic supplementary material, appendix S3.

### (b) Calculating migration distance

Individuals were assumed to be at their main wintering locations between January and February, when GPS data indicates that 95% of lesser black-backed gulls are within their southernmost region (see ‘Determining winter resighting range’ and electronic supplementary material, figure S1). Not all ringed birds were resighted every year. However, GPS data indicates that individuals have a high winter area fidelity and thus migratory distance is highly repeatable within individuals (*R* = 0.81, 0.57–0.93 95% CI, *n* = 77 [19]) so we assumed winter location was similar across years. Therefore, for birds of which the wintering location was known in multiple years, the median latitude and longitude of all resightings or GPS data between January and February, pooling across all years, was used as an individual's winter location. Since Africa is the furthest region but had low-resighting probabilities, we included observations in December in Africa to determine the winter location. This added six individuals that winter in Africa to the dataset, five of which were resighted in West Africa, and were therefore unlikely to still be migrating. One individual is known to have changed wintering region during the period of this study and was removed from further analysis. Migration distance was calculated as the geodesic between the breeding colony and their median winter location (implemented in the geodist package in R [38]).

### (c) Reproductive parameters

We visited the Texel breeding colony every third day throughout the breeding season (April–July) to mark new nests, number and measure length and width of newly laid eggs (in cm, to the mm), and identify parents. Nests were followed until hatching (see [39] for more details). Clutches mostly consist of three eggs, of which the last egg is often smaller than the first two [33]. Laying date refers to the laying date of the first egg in the clutch. The pre-laying period is defined as the period between the arrival date within the colony and the laying date. Relative laying date is the deviance between an individual's laying date and the median laying date of the lesser black-backed gull colony each year (data in [33], electronic supplementary material, table S1). Egg volume (cm^3^) was calculated as 0.5035 × length × width^2^ [40]. We included reproductive data from individual gulls from the year they were captured and marked (i.e. with colour rings and GPS tags if applicable), as well as any subsequent season where reproductive monitoring was carried out for those individual's nests. Nests in IJmuiden were not followed in the same detail as in the colony on Texel, so the IJmuiden individuals were not used for analyses of reproductive efforts, only for survival analysis.

### (d) Data analyses

We first tested whether migration distance was related to the number of days between arrival and laying (pre-laying period) with a generalized mixed-effect model (GLMM) with individual as a random effect. For this analysis, we only used GPS-tagged individuals because we needed to know exact arrival dates. Then, to examine parameters linked to reproduction, we fitted GLMMs of the relative laying date, mean egg volume, volume ratio between first and third egg, and hatching success (proportion of eggs laid that survived until hatching) as a function of migration distance. The ratio between first and third egg was used as a measure of reproductive investment as the third egg is often smaller than the first egg in a clutch [33]. Mean egg volume and ratio between the volume of the first and third egg were only modelled for females. In the analyses examining relative laying date and hatching success we included sex, and the interaction between migration distance and sex. Individual was always included as a random effect, and year was included as a random effect when analysing egg volumes and hatching success. Migration distances of both parents were known for 10% of the nests, but including nest ID as random effect in the models to account for pseudo-replication resulted in a singular fit. We therefore randomly excluded one of the paired parents from the analysis. In the electronic supplementary material, appendix S3 we show, by iterating this process 500 times, that this had no effect on the outcome. Mean egg volume was only calculated for clutches with three eggs, and only using the volumes of the first three eggs if number of eggs laid exceeded three (if eggs are predated, females occasionally produce replacement eggs or repeat clutches). We included relative laying date in the models for egg volumes and hatching success as laying date has shown to be related to these variables [33]. Gaussian error distributions were used for all models except proportion of eggs hatched, which was modelled with a binomial distribution with a logit link function. Models were validated by visually inspecting histograms of residuals, and scatter plots of residual versus fitted values and migration distance. Sample sizes are reported in table 1, and data are published online [41]. We used likelihood ratio tests to compare models with and without migration distance. GLMMs were modelled in R using package lme4 [42].

**Table 1.** Parameter estimates (± s.e.) of GLMMs estimating the effect of migration distance on pre-laying period, relative laying date (deviation from year-specific median laying date), mean egg volume, ratio between third and first egg volumes and proportion of eggs hatched. Significant effects are shown in italics. Migration distance had only a significant effect in the model with pre-laying period as response variable. The sample size for relative laying date and proportion hatched are after randomly selecting one individual from a nest of which the migration distance of both partners was known.

	pre-laying period (days)		relative laying date (days)		mean egg volume (mm^3^)		third : first egg ratio		proportion hatched	
**fixed effects**										
intercept	62.28 ± 4.59		−1.93 ± 0.72		74.64 ± 1.39		0.930 ± 0.012		1.15 ± 0.27	
migration distance^a^	**−***8.88 ± 2.13*		0.25 ± 0.39		−0.53 ± 0.77		−0.002 ± 0.006		0.04 ± 0.14	
sex			0.40 ± 0.76						−0.27 ± 0.29	
sex × migration distance^a^			−0.05 ± 0.80						0.29 ± 0.29	
relative laying date					−0.01 ± 0.18		**−***0.003 ± 0.001*		**−***0.07 ± 0.03*	
**random effects**										
individual	58.92		7.92		26.32		1 × 10^−3^		1.21	
year					1.96		7 × 10^−5^		0.32	
residual	109.81		19.38		11.31		2 × 10^−3^		-	
**sample size**										
no. of nests	34		280		105		105		281	
no. of individuals	4 females, 15 males		65 females, 81 males		64 females		64 females		67 females, 77 males	
**log-likelihood test**^b^	*χ*^2^	*p*	*χ*^2^	*p*	*χ*^2^	*p*	*χ*^2^	*p*	*χ*^2^	*p*
migration distance	*12.39*	*0.0004*	0.43	0.51	0.47	0.50	0.06	0.77	0.07	0.80
sex			0.29	0.59					1.08	0.30
sex × migration distance			0.004	0.95					0.97	0.32
relative laying date					0.007	0.93	*4.45*	*0.03*	*6.35*	*0.01*

^a^Migration distance in 10^3^ km.

^b^d.f. is 1 in all cases.

We estimated adult apparent survival (Phi) and resighting probability (*p*) from ring resightings of gulls using Cormack–Jolly–Seber mark–recapture models in program MARK [43] using RMark [44]. For individuals with GPS tags, only ring resightings and not GPS tracks were included as our GPS devices only download data in the colony. Therefore, if data were not downloaded in a subsequent year and the bird was not resighted, we could not determine whether it had actually died (and during which period), if the GPS had stopped working or if the bird had dispersed to a different colony.

For the mark–recapture models, we used two resighting periods during the year: May–July (during breeding) and December–February (in winter). Survival is estimated for the interval between the resighting periods; therefore, the survival probability between winter and breeding represents ‘spring survival’ and the survival between summer and winter ‘autumn survival’. We account for unequal time steps in the models, and survival is estimated as a half-yearly survival probability. Individuals marked as fledglings could only enter the dataset as adults (more than 5 years old) to exclude age-dependent effects. We analysed a set of models where survival probability was constant throughout the year (constant), differed with migration distance (distance), differed by season (season), where migration distance influences spring survival only (spring × distance + autumn), where migration distance influenced autumn survival only (spring + autumn × distance) and where migration distance influenced survival differently per season (spring × distance + autumn × distance). For each model, resighting probability in winter could differ between wintering destinations (France/UK, Iberia, Africa) due to differences in resighting efforts. In summer, resighting probability may depend on whether a bird was marked as an adult in IJmuiden or on Texel, or marked as a fledgling. This is because resighting effort is expected to be higher in IJmuiden than on Texel, while fledglings may disperse from the natal colony as adults, lowering summer resighting probability. We did not let survival or resighting probability vary over time or by sex, due to the relatively small sample size. Number of individuals included in this analysis are France / UK = 98, Iberia = 208, Africa = 51.

Goodness-of-fit is tested with U-Care [45] called from R with R2Ucare [46]. The overall fit, which was tested separately for birds ringed in IJmuiden, Texel or as fledglings and then summed, was not significant (*χ*^2^ = 129.1, d.f. = 153, *p* = 0.92). Model selection was based on Akaike's information criterion adjusted for small sample sizes (AICc) [47]. We also present model-averaged results, where predicted survival from each model in the set are averaged, weighing by the Akaike weights (*w*_i_) [47].

### (e) Animal ethics

We followed the Dutch Animal Welfare Act Articles 9, 10 and 11 of animal experiment documents and worked under licence number AVD8020020174225 to handle and tag lesser black-backed gulls. The GPS tags plus harnesses were always below 3% of their body mass and were adjusted to the individual. Ringing was done under legislation from the Dutch Ringing Centre (licence number E52 and 392) and always carried out by a certificated ringer.

## 3. Results

**Figure 1.**
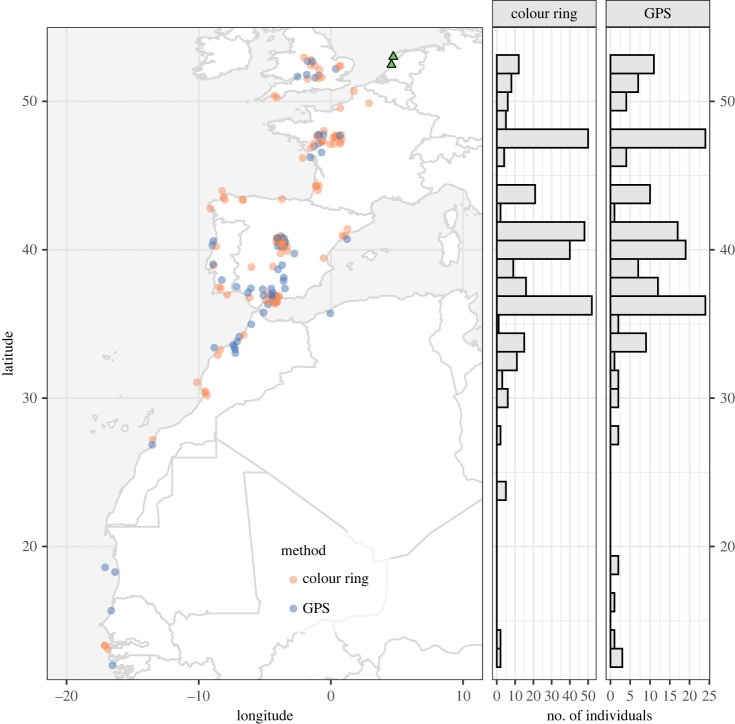
Map of wintering locations of lesser black-backed gulls breeding in two colonies in the Netherlands (green triangles). Individuals are coloured to show whether we know their wintering location based on colour-ring sightings (orange) or GPS locations (blue). Points were jittered in both directions to increase visibility of overlapping points. Histograms on the right show the latitudinal distribution of winter locations determined from colour-ring resightings and GPS.

**Figure 2.**
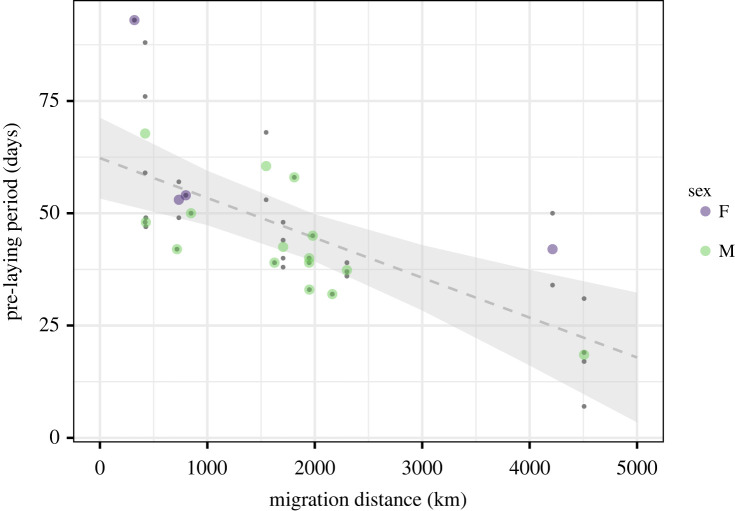
Pre-laying period, the number of days between arrival in the colony and laying of the first egg, has a negative relationship with migratory distance (grey dashed line). Large points are the mean values of pre-laying periods for individuals (*n* = 19) of which we have multiple years of GPS data; small points represent per year pre-laying periods per individual (*n* = 34). For those individuals of which we only had data for 1 year, the large and small point overlap. The grey area shows the 95% CI. Due to the small sample size for females, we did not include sex in the analysis.

**Figure 3.**
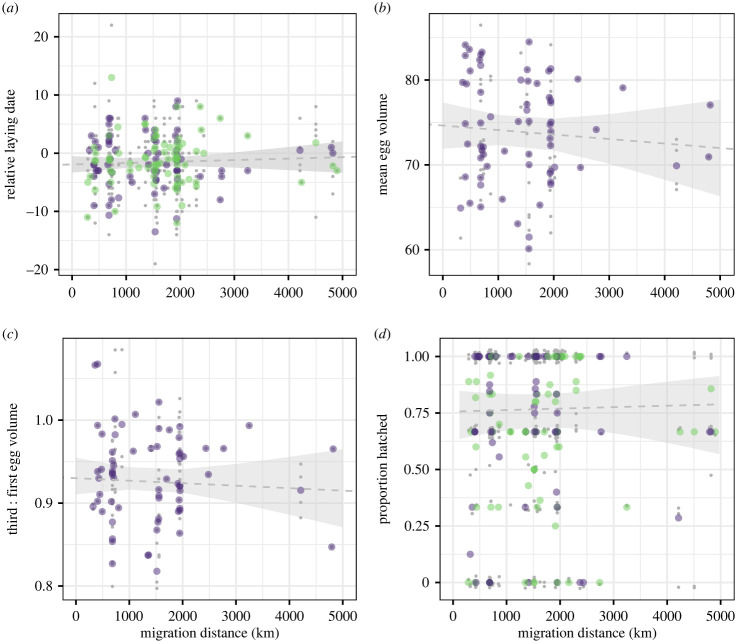
(*a*) Relative laying date, (*b*) mean egg volume, (*c*) ratio between third and first egg volumes and (*d*) proportion of eggs hatched by female (purple) and male (green) lesser black-backed gulls in relation to their migration distance. Large points are the mean values of birds with multiple years of data; small grey points are all data points (thus multiple data points per individual). Small points were jittered vertically in (*d*) so overlapping values are visible. Grey dashed lines show the slope; however, note that effect of migration distance was not significant for any reproductive parameter (*p* > 0.4). The grey area around the lines show the 95% CI.

Migration distances between individuals differed by more than 4500 km (range 279–4898 km, median 1537), with Iberia being the most common winter region based on both resightings and GPS data (figure 1). Duration of the pre-laying period decreased with migration distance (*p* < 0.001; figure 2, table 1), ranging from 93 days for an individual that wintered in the UK to 7 days for an individual that wintered in Western Africa. We did not find a correlation between migration distance and relative laying date (*p* = 0.51; figure 3*a*, table 1), suggesting that regardless of variation, the duration of the pre-laying periods was sufficient to synchronize laying dates. There was no evidence of a relationship between migration distance and egg volume (*p* > 0.5; figure 3*b*, table 1), the ratio between the volume of the first and third egg (*p* > 0.5; figure 3*c*, table 1), or proportion of eggs hatched (*p* > 0.4 for both sexes; figure 3*d*, table 1). Sex and the interaction between sex and migration distance did not explain variation in laying dates (*p* > 0.5) nor hatching success (*p* = 0.3) (table 1). We did find a relationship between relative laying date and the ratio between the volume of the first and third egg (*p* = 0.03) and hatching success (*p* = 0.01) (table 1).

**Figure 4.**
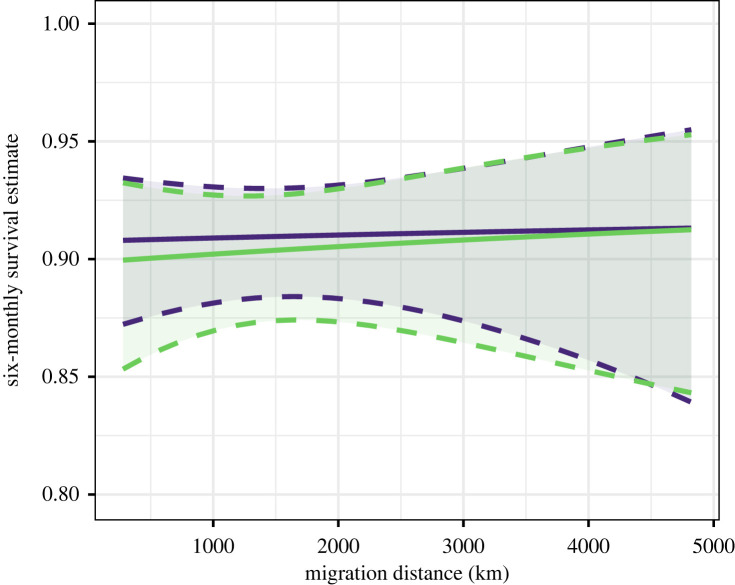
Model-averaged apparent survival estimates with 95% confidence intervals of lesser black-backed gulls by season (autumn = purple, spring = green) and migration distance (based on all models in table 2).

The best-supported survival model had a constant survival parameter, and thus there was no support for an effect of migration distance on either overall apparent survival or survival during one or both migratory seasons (table 2). The six-month survival probability was estimated as 0.91 (0.90–0.93 95% CI; table 3). The second- and third-best-supported models were around 2 AICc from the top model, but had one additional parameter and similar log-likelihoods of 3618.3 and were therefore not considered competitive [47,48]. Additionally, the 95% confidence interval of these parameters overlapped zero (autumn: −0.65–0.77; migratory distance: −0.20–0.22), also shown by the model-averaged results (figure 4). Resighting probability during summer was higher than during winter, and highest for birds marked in IJmuiden. Resighting probability during winter was lowest for birds wintering in Africa (table 3).

**Table 2.** Model selection results of lesser black-backed gull survival probability (Phi), where we examined whether survival probability was influenced by season and migration distance, including different distance effects per season. Models are ordered by *Δ*AIC_c_, where no. of par. is the number of parameters, *w*_i_ is the Akaike weight and deviance is the residual deviance. An interaction between two parameters is indicated with × .

model	no. of par.	AICc	*Δ*AICc	*w*_i_	deviance
Phi(constant)	7	3632.411	0.000	0.481	2585.603
Phi(season)	8	3634.404	1.994	0.178	2585.576
Phi(distance)	8	3634.427	2.016	0.176	3618.336
Phi(spring × distance + autumn)	9	3636.335	3.924	0.068	3618.221
Phi(spring + autumn × distance)	9	3636.398	3.988	0.065	3618.285
Phi(spring × distance + autumn × distance)	10	3637.774	5.364	0.033	3617.635

**Table 3.** Parameter estimates (± s.e.) and 95% confidence intervals of the most parsimonious model for six-month apparent survival (Phi) with resighting probability (*p*) of lesser black-backed gulls.

parameter	estimate	95% confidence interval
*Phi* (constant)	0.914 (0.006)	0.901–0.925
*p* (marked as fledgling)^a^	0.240 (0.033)	0.181–0.309
*p* (marked on Texel)	0.667 (0.022)	0.622–0.710
*p* (marked in IJmuiden)	0.875 (0.015)	0.843–0.902
*p* (FRUK)	0.240 (0.033)	0.181–0.309
*p* (IB)	0.420 (0.020)	0.381–0.459
*p* (AFR)	0.280 (0.049)	0.194–0.384

^a^marked as fledgling but only entered the dataset as adult.

## 4. Discussion

Examining the consequences of different migration strategies on reproductive performance and survival is central to understanding population dynamics and evolution of migratory systems [49,50]. Individual lesser black-backed gulls that migrate furthest arrived latest in the breeding colony [19]. In this study, we found that lesser black-backed gulls that migrate further had a shorter pre-laying period than shorter-distance migrants, rather than having later laying dates relative the rest of the colony. Surprisingly, this did not carry over to impact subsequent reproductive parameters such as egg volumes or hatching success as we had predicted. We also found no effect of migration distance on apparent survival.

In colonially breeding species, laying dates are often synchronized and breeding before or after the peak results in lower reproductive success [51–53]. In our study colony, late breeders had smaller third eggs compared to first eggs, lower hatching success and four times lower fledging success compared to early and peak laying birds [33], which may explain why late-arriving long-distance migrants did not postpone breeding. Even though the pre-laying period was exceptionally short for one male (7 days), he apparently arrived in time to breed. Laying date therefore seems to be a stronger driver for hatching success than the potential penalties for a short pre-laying period, such as occupying a low-quality territory or perhaps mating with a low-quality partner. However, we cannot account for variation in breeding propensity between strategies; there is a possibility that birds who arrive too late to synchronize with the laying peak in a certain year, either because they arrive after the laying peak, cannot establish a territory or find a mate in time, or arrive with too low energy stores to produce eggs, forego breeding.

Egg production is energetically demanding [54]. Bolton *et al*. [55] showed with an experiment that in a year when lesser black-backed gulls were energetically constrained, those that were supplemented with food before egg laying laid larger eggs. Nevertheless, female lesser black-backed gulls who migrated far and consequently had a shorter pre-laying period to replenish their energy stores seemed to have sufficient energy to equally invest in egg volume compared to gulls which wintered close to the colony. Perhaps lesser black-backed gulls migrating long distances are not energetically constrained upon arrival. Our previous work showed that on an annual basis, long-distance migrants travelled as many kilometres as short-distance migrants [9] and migration strategy did not influence their annual energy expenditure, though African migrants have higher rates of energy expenditure during spring than migrants with other strategies [32]. It is also possible that lesser black-backed gulls which migrated furthest stayed in better quality winter areas and therefore were able to store enough energy to compensate their migratory flight [20]. Alternatively, the short pre-laying period is sufficient to recover from long-distance migration and prepare for egg laying.

We could not detect a relationship between migration distance and survival probability in lesser black-backed gulls. Instead, our results suggest that survival was constant across seasons and regardless of migration distance. For long-lived species even a small variation of adult survival probability may have consequences for fitness, we should therefore be careful with our interpretation. Yet, our result corresponds with our study showing that annual energy expenditures are similar among migration strategies, because energetic costs of migration are offset by slightly lower activity costs during winter and breeding periods, though it remains unknown whether energy intake differs among winter areas [32]. In many avian species [56], including this species [32], spring migration is condensed into a shorter time period than autumn migration, and therefore often more energetically demanding and thus potentially more risky. If the spring migration period has a higher mortality risk, increased migration effort during spring should lead to elevated mortality relative to autumn, as found in a raptor [12], a songbird [57] and a swift [58], and we would expect this effect to be increased as migration distance increases as seen in a wading bird [14]. A lack of seasonal effects in our survival model, for even the longest-distance migrants, could be indicative that mortality is not significantly higher during any migratory stage in our system.

Elevated mortality during migration is often attributed to food limitation at stopover sites [59,60] and severe weather (reviewed in [61]), which may be exacerbated before or during the crossing of geographical barriers where no suitable habitat is available for landing during emergencies [12,14,62]. However, lesser black-backed gulls, being able to rest and forage on both land and at sea, and detour around arid inland areas [19], do not cross any major geographical barrier along their migration routes and thus may be less susceptible to these hazards typically associated with migration. Further, survival costs of certain migration strategies may only exist in years with severe climatic conditions [63,64]. Due to a limited sample size, we could not include year variation in our models, making detection of these weather-dependent costs difficult.

Coexistence of different migration strategies within a population is thought to evolve and be maintained either as a result of equal fitness payoffs across strategies, typically modelled as evolutionarily stable strategies [65]. Alternatively, the optimal strategy for an individual is conditional on characteristics of individual (e.g. subordinate individuals are not sufficiently competitive to remain near breeding areas and thus ‘make the best of a bad job’ by migrating to less competitive areas [66–68]). Most empirical research supports the latter (reviewed in [69]). In this study, however, we found no effect of winter region on either survival or reproductive parameters, suggesting there is no strong selective pressure acting on migration strategy within these populations once individuals reach breeding age. The question remains how these different migration strategies for lesser black-backed gulls have developed or whether the migration strategies of lesser black-backed gulls have changed over the past decades due to climate change or shifts in (anthropogenic) food availability [14,70].

Given that differential migration is a widely spread phenomenon within migratory species [3,69], encompassing species that differ greatly in fundamental aspects of their ecology and physiology such as breeding habits, foraging ecology and habitat requirements, morphology, thermal tolerance and cognitive abilities, it is perhaps unsurprising that empirical evidence of the fitness consequences of different migration strategies are inconsistent. Thus, the general assumption that migrating far is costly and has consequences on survival and reproduction does not apply universally to all migratory species, which probably contributes towards the huge diversity in migration strategies that exist among species, populations and individuals.

## Data Availability

Data associated with this manuscript are archived in the Data Archive System (DAS) of NIOZ Netherlands Institute for Sea Research and publicly available at https://dataportal.nioz.nl/doi/10.25850/nioz/7b.b.td [41]. The data are provided in the electronic supplementary material [71].
